# Uncertainty in REDD+ carbon accounting: a survey of experts involved in REDD+ reporting

**DOI:** 10.1186/s13021-024-00267-z

**Published:** 2024-07-27

**Authors:** Brett J. Butler, Emma M. Sass, Javier G. P. Gamarra, John L. Campbell, Craig Wayson, Marcela Olguín, Oswaldo Carrillo, Ruth D. Yanai

**Affiliations:** 1 Northern Research Station, USDA Forest Service, Amherst, MA USA; 2 Department of Environmental Conservation, University of Massachusetts Amherst, Amherst, MA USA; 3 Forestry Division, Food and Agriculture Organization of the United Nations, Rome, Italy; 4 Northern Research Station, USDA Forest Service, Durham, NH USA; 5 International Programs, USDA Forest Service, Washington, DC USA; 6 SilvaCarbon, Climate Change Unit, USDA Forest Service International Programs, Mexico City, Mexico; 7 Department of Sustainable Resources Management, State University of New York College of Environmental Science and Forestry, Syracuse, NY USA

**Keywords:** REDD+, Carbon credits, Tropical deforestation, Forest carbon, Survey

## Abstract

**Background:**

Reducing Emissions from Deforestation and forest Degradation (REDD+) is a program established under the United Nations Framework Convention on Climate Change (UNFCCC) to reduce carbon emissions from forests in developing countries. REDD+ uses an incentive-based approach whereby participating countries are paid to reduce forest carbon loss and increase carbon storage. Country-level carbon accounting is challenging, and estimates of uncertainty in emission reductions are increasingly required in REDD+ reports. This requirement is hard to meet if countries lack the necessary resources, tools, and capabilities. Some REDD+ programs adjust their payments for the uncertainty reported, which presents a perverse incentive because uncertainties are larger if more sources of uncertainty are reported. We surveyed people involved in REDD+ reporting to assess current capacities and barriers to improving estimates of uncertainty.

**Results:**

Representatives from 27 countries (44% of REDD+ countries at the time of survey implementation) responded to the survey. Nearly all respondents thought it important to include uncertainty in REDD+ reports, but most felt that the uncertainty reporting by their countries was inadequate. Our independent assessment of reports by these countries to the UNFCCC supported this opinion: Most countries reported uncertainty in activity data (91%) but not in emission factors (4–14%). Few countries use more advanced approaches to estimate uncertainty, such as Monte Carlo and Bayesian techniques, and many respondents indicated that they lack expertise, knowledge, or technical assistance. Other barriers include lack of financial resources and appropriate data. Despite these limitations, nearly all respondents indicated a strong desire to improve estimates of uncertainty in REDD+ reports.

**Conclusions:**

The survey indicated that people involved in REDD+ reporting think it highly important to improve estimates of uncertainty in forest carbon accounting. To meet this challenge, it is essential to understand the obstacles countries face in quantifying uncertainty so we can identify where best to allocate efforts and funds. Investments in training and resources are clearly needed to better quantify uncertainty and would likely have successful outcomes given the strong desire for improvement. Tracking the efficacy of programs implemented to improve estimates of uncertainty would be useful for making further refinements.

**Supplementary Information:**

The online version contains supplementary material available at 10.1186/s13021-024-00267-z.

## Background

Deforestation and forest degradation is the second largest source of carbon emissions to the atmosphere after the energy sector [1]. Therefore, forest conservation, forest establishment and growth, and sustainable forest management are key mechanisms for mitigating global climate change [2, 3]. Forests store vast amounts of carbon, but their behavior as sinks [4] or sources [5] is uncertain. To enhance carbon sequestration and storage in these ecosystems, a results-based program was established under the United Nations Framework Convention on Climate Change (UNFCCC) to reduce emissions and increase forest carbon stocks: REDD+ (Reducing Emissions from Deforestation and forest Degradation “plus” the sustainable management of forests and the conservation and enhancement of forest carbon stocks). To participate in REDD+, countries must be considered economically developing and must implement mitigating actions and report the consequent reductions in carbon emissions [6]. Payments for reducing carbon emissions are made to these countries through market and non-market incentives by public sector finance mechanisms, such as the Green Climate Fund (UNFCCC) and the Forest Carbon Partnership Facility (FCPF), collaborative international partnerships (e.g., Central African Forest Initiative), bilateral agreements (e.g., Norway, Germany, UK, Japan’s Joint Crediting Mechanism), internal national governments (e.g., Colombia), private corporations (e.g., PetroBrás), and newer public–private initiatives (e.g., Leaf Coalition).

It is difficult enough to quantify country-level forest carbon stocks; carbon emissions and sequestration are even more challenging to estimate because they are based on the rate of change of carbon stocks [7]. The REDD+ program compensates countries for reducing net carbon emissions, which is determined by comparing estimates of carbon emissions and sequestration during a reference period with the period for which emission reductions are credited [8]. Because of the difficulty in quantifying carbon fluxes, uncertainty in estimates of emission reductions can be very high. In fact, when emission reductions are small, uncertainties can exceed 100% of the reductions [9], meaning that it is not clear whether emissions have in fact been reduced or increased. The risk associated with this uncertainty can be mitigated by discounting payments for emission reductions [7, 10].

In countries without extensive forest inventory programs, carbon emission reductions can be calculated from “activity data” and “emission factors” [11, 12]. Activity data might be based on information about logging removals of carbon, but are often based on map-based assessments of the rate of change in land area in forest (e.g., deforestation and afforestation) and changes in the area of forest degradation, over a given period (e.g., number of hectares per year). In this case, emissions for the country can be calculated by multiplying these “activity data” by the difference in carbon stocks per unit area for each land use transition, known as “emission factors” [13].

**Fig. 1:**
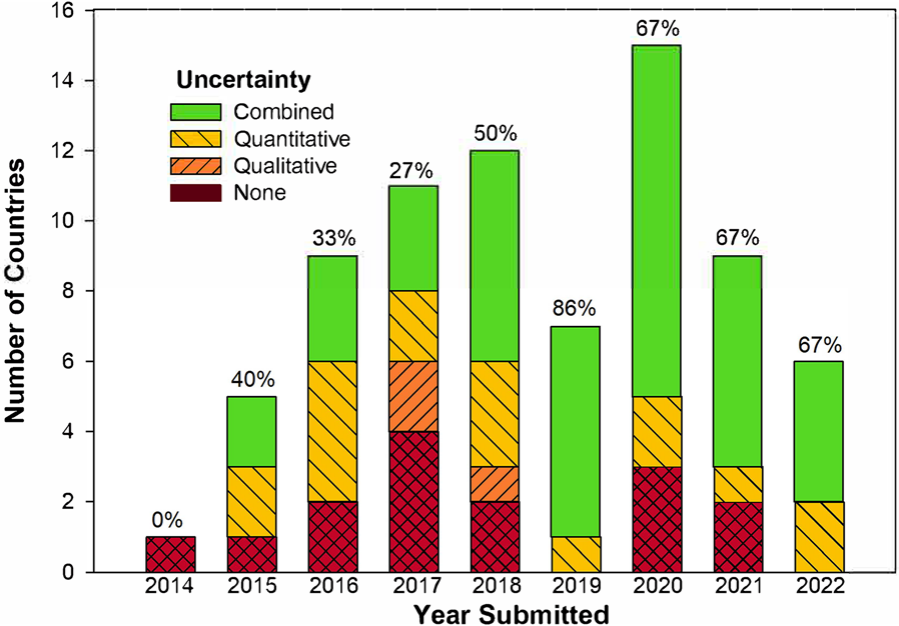
As of 2022, 56 countries, representing most of the forest area in developing countries, reported reference levels of REDD+ emissions for technical assessment under the UNFCCC, with some countries submitting more than once. Numbers show the percentage of submissions that included a combined (not necessarily complete or correct) accounting of uncertainty, as opposed to no reporting of uncertainty, some qualitative discussion but no quantification of uncertainty, or some quantification of uncertainty sources without an estimate of uncertainty in emissions

From 2014 to 2022, 56 countries, representing most of the forest area in developing countries, reported reference levels of REDD+ emissions for technical assessment under the UNFCCC, with increasing attention to uncertainty (Fig. 1). REDD+ activities reported to the UNFCCC claim to have reduced emissions by 13.7 billion tCO2e [14]. Needless to say, correctly quantifying uncertainty is essential for assessing confidence in these reported reductions of carbon emissions [15].

Quantifying uncertainty requires technical expertise in error propagation, which is currently lacking, as shown by some countries reporting combined errors an order of magnitude smaller than the individual sources of error [7]. Uncertainty in carbon estimates has taken an increasingly important role in international reporting because it affects payments made for emission reductions under the different funding schemes available [7, 16]. Specifically, the reported uncertainty affects the payment a country can receive for carbon mitigation actions [17].

To assess awareness and knowledge about uncertainty in REDD+, we conducted a survey of experts involved in REDD+ reporting to better understand their views on uncertainty in carbon accounting, including their perception of the barriers they face in improving uncertainty estimates. The goal of this survey was to identify existing information gaps related to comprehensive and accurate estimation of uncertainties, assess current needs to build capacity, and enable informed decision making to improve monitoring and ultimately reduce uncertainty in emission reductions reports.

## Methods

The survey was implemented using the online platform Qualtrics. The survey and outreach materials were translated into Spanish and French, using DeepL and verified by native speakers, to cover the most common languages spoken by REDD+ country reporters. The first question on the survey asked respondents to select their language, and the rest of the survey was presented in that language. Responses were merged across languages for summary and analysis.

The target population for the survey was people involved in reporting REDD+ carbon accounting through report preparation, technical assistance, oversight, or reviewing. The sampling frame included all individuals listed as focal points on REDD+ reports on the UNFCCC web site (https://redd.unfccc.int/) as of June 2022 and was augmented with experts known by the authors to be involved in REDD+ reporting. This list included individuals from country governments, non-governmental organizations, international organizations, private or independent consultants, and academic institutions. Survey participants were asked to complete the survey for the REDD+ report and country that they worked on most recently. The sampling frame was as complete as possible to reach the maximum number of relevant experts, as the total number of people involved in REDD+ reporting is relatively small; we identified 182 individuals. Survey implementation and questionnaire design followed the method outlined by Dillman et al. (2014), which involved sending the questionnaire as a personalized link through email, with up to two reminder emails for people who did not complete it. The survey was disseminated between May and July 2022, following review and approval of survey questions and procedures by the University of Massachusetts Amherst Institutional Review Board (approval #2701).

Topic areas covered on the survey included general level of uncertainty reporting in country reports along with specific sources of error, experience with specific uncertainty quantification methods (analytical error propagation, Bayesian inference, and Monte Carlo simulation), assessment of knowledge associated with uncertainty quantification based on provided scenarios and multiple choice responses, barriers to and potential opportunities for improving quantification and reporting, and general background information about the respondents (e.g., education level and affiliation). The specific sources of uncertainty related to emission factors asked about were sampling error, measurement error, error in root-to-shoot ratios, and uncertainty in biomass models. Uncertainty in activity data was treated as a single source, because a vast majority of countries report only sampling error for activity data. Brief descriptions and examples were provided for each source of uncertainty. The full questionnaire is available in Appendix 1.

Of the 182 individuals from 65 countries who were invited to participate in the survey, 33 had incorrect or outdated contact information. The number of valid responses received was 49, yielding a cooperation rate of 33%. The number of countries represented by these responses was 27 or fewer, depending on the question (not all respondents answered all the questions). There were also responses from 7 additional institutions (e.g., FAO, UNFCCC).

As with all surveys, there are potential sources of error that need to be considered and minimized [18]. Potential coverage error issues were mitigated by including all publicly listed report contributors and augmenting the sampling frame with other known actors. Sampling error, per se, was not an issue because all members of the target population who could be identified were invited to participate (i.e., no sampling occurred). Four cognitive interviews were conducted with members of the target population. These were used to improve the wording of the questions but not to generate results for analysis. The 33% cooperation rate, while not unreasonable for a survey and with good coverage across REDD+ countries, suggests the potential for nonresponse bias. Because of the small population size, no nonresponse bias assessments could be conducted and the potential for nonresponse bias should be considered when interpreting the results. It seems likely that people contacted who had a greater interest in or knowledge of uncertainty quantification would have been most likely to respond.

Selected results are presented below in terms of countries or individuals, depending on the scope of the question. For country statistics, modal values were calculated for countries with more than one respondent and ties were broken by taking the highest value. In calculating percentages, non-respondents to specific questions (i.e., NA values) were dropped. Polychoric correlations [19] were used to quantify relationships involving ordinal variables and *Χ*^*2*^ tests were used to test for relationships between categorical variables.

To assess the accuracy of the responses regarding what sources of uncertainty were reported, we independently assessed the UNFCCC reports from the 23 countries represented in this aspect of our survey [20]. We evaluated the accuracy of error propagation in the reports and we scored whether each country reported sampling error, measurement error, errors in root-to-shoot ratios, uncertainty in biomass models and uncertainty in activity data.

## Results

**Fig. 2:**
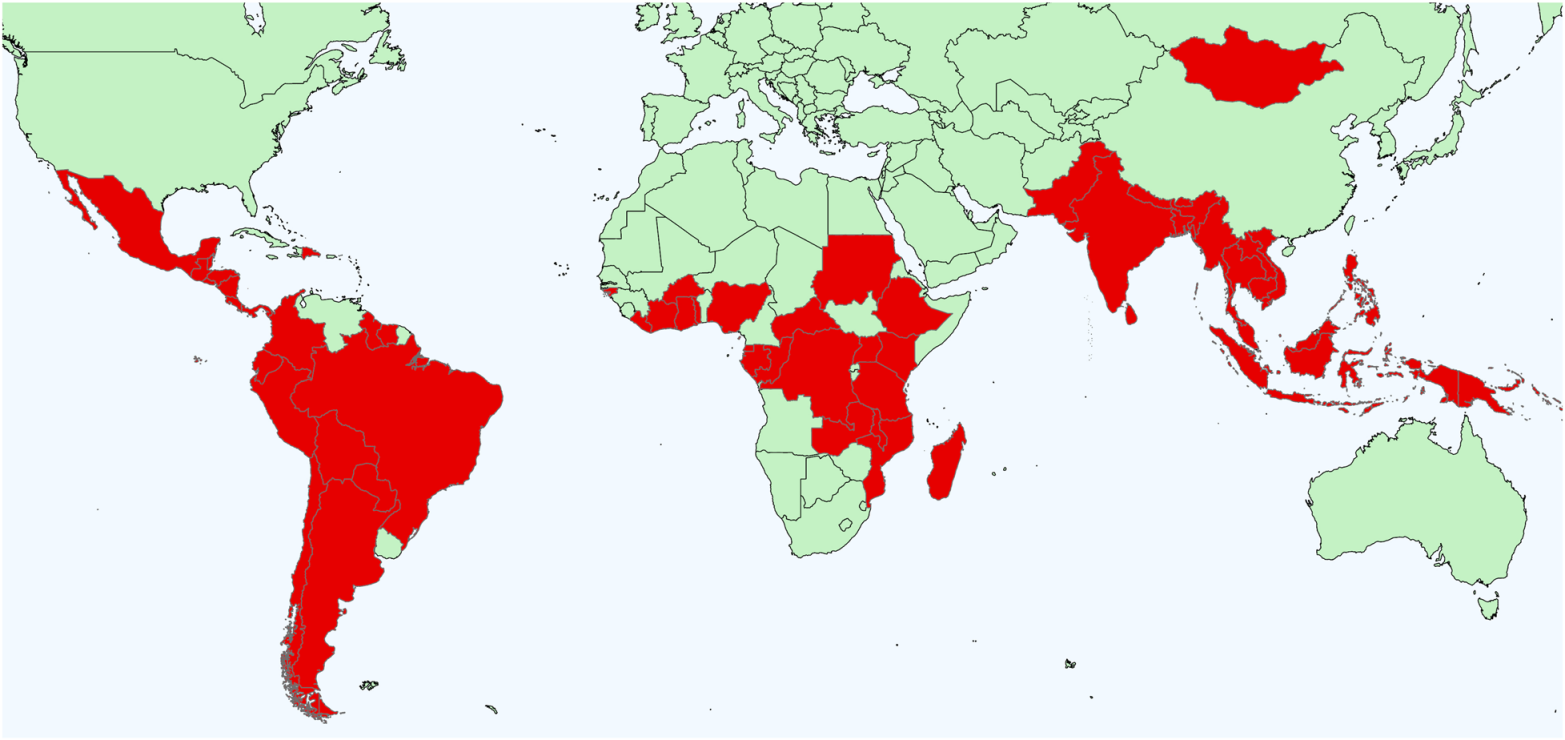
Map of countries actively participating in REDD+ as of 2023. Representatives from 27 countries responded to the survey (41% of REDD+ countries at the time it was distributed). Specific countries that responded are not indicated on the map to protect anonymity, and not all countries shown were participating in REDD+ at the time the survey was distributed

Responses were received from 49 individuals representing 27 of the 61 countries (44%) with REDD+ reports at the time of survey implementation (i.e., May through July 2022). The countries that responded were reasonably well distributed across the REDD+ regions, with 9 countries in the Latin America-Caribbean region, 5 in the Asia–Pacific region, and 13 in Africa (Fig. 2).

Most of the individuals who responded (73%) worked for government agencies, 10% were employed by international organizations, 10% worked for academic or research institutions, and 7% worked for private organizations or other groups. Roles in the reports included preparation (64%), technical assistance (45%), reviewing (36%), and oversight (20%). These percentages sum to more than 100 because respondents could have multiple roles: 41% had two or more, with the most common combination being “preparation and technical assistance” and “technical assistance and reviewing.” In terms of formal education, all had at least some college experience and 60% had a graduate degree.

Uncertainty for REDD+ reporting was rated as “extremely important” or “very important” by 95% of the responding individuals. One individual responded that it was “somewhat important,” another that it was “a little important,” and no one responded that it was “not at all important.”

Compared to its perceived importance, uncertainty reporting was low, especially for sources involved in emission factors (Table 1). Our independent evaluation of reports by the 23 countries represented in this aspect of the survey found uncertainty in activity data to be the source most commonly reported (91% of countries); respondents to the survey agreed that uncertainty in activity data was commonly reported (74% of countries). For sources of uncertainty in emission factors, sampling error was the most commonly reported (87% of countries, according to our evaluation of reports, and 57% according to survey respondents). Other sources of uncertainty in emission factors were rarely reported–we found rates ranging from 4 to 14% for root-to-shoot ratios, biomass models, and measurement error; survey respondents were more optimistic about reporting rates (26–48%) for these sources. It’s interesting that these sources were rated highly in importance (68–77% of respondents rated them as very or extremely important on a 5-point scale), though not quite as highly as sampling error (84% for emission factors and 80% for activity data–generally the only source reported in activity data is the map sampling error), while self-reported experience levels with all sources were low, with only 36–61% being very or extremely experienced, depending on the source (Table 1).

**Table 1:** Levels of reporting, experience, and importance for improvement for sources of error related to uncertainty of emission factors and activity data in REDD+ reporting. Uncertainties in activity data were not disaggregated into multiple sources because a vast majority of countries report only sampling error for activity data

Source	Our assessment	Survey results		
	Countries reporting (%)	Countries reporting (%)	Individual’s experience levels^a^ (%)	Individual’s importance ratings^b^ (%)
Emission Factors				
Sampling error	87	57	61	84
Measurement error	4	35	50	75
Error in root-to-shoot ratios	13	26	36	68
Uncertainty in biomass models	14	48	45	77
Activity Data				
Uncertainty in activity data	91	74	45	80

^a^Individuals who rate themselves as extremely or very experienced with a source or error on a 5-point Likert-scale

^b^Individuals who rate a source of error as extremely or very important on a 5-point Likert-scale

Respondents gave low scores for comprehensiveness and correctness of REDD+ uncertainty reporting, as did we, in our independent assessment of their reports. They indicated that 19% of countries have reported all five sources of error included in Table 1 (we found 4%), 59% reported most sources of error (we found that 13% reported 3 or 4 sources), 18% reported some or a few sources of error (we found that 83% reported 1 or 2), and no countries reported no sources of error, but the response was “don’t know” for one country. The uncertainty reporting was rated by the respondents as highly or mostly correct for 56% of the countries, moderately correct for 26% of the countries, a little or not at all correct for 8% of the countries, and the response was “don’t know” for 11% of the countries. In our evaluation of the 23 reports represented by survey responses, we judged 17% of them to be correct.

Notably, perceptions of the comprehensiveness or correctness of uncertainty reporting varied across respondents for the same country. Nine countries had two or more responses to this survey. In terms of completeness, the respondents for 1 of these 9 countries reported the same levels, 6 countries differed by only one category, and 2 countries had responses with lower agreement. In terms of correctness, 3 countries reported identical levels, 4 countries reported differences of one category, and 2 countries (not the same as the ones for completeness) had lower agreement. Thus, some of the discrepancy between our perceptions and theirs may be due to variability in expertise or experience among respondents.

**Fig. 3:**
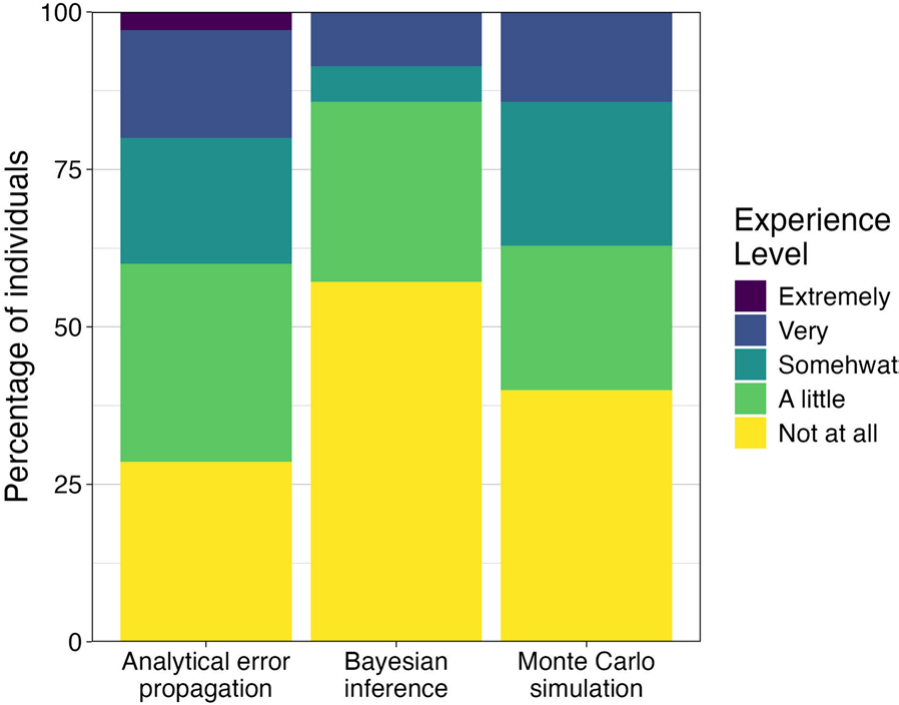
Levels of experience with approaches to error propagation for estimating uncertainty in REDD+ reporting

Survey respondents reported low experience levels for all of the techniques used for error estimation (Fig. 3). Eighty-six percent of individuals had little or no experience with Bayesian inference, 63% had little or no experience with Monte Carlo simulation, and 60% had little or no experience with analytical error propagation. Correlations across the techniques were relatively high: ρ = 0.66 between analytical error propagation and Monte Carlo simulation, ρ = 0.63 between Monte Carlo simulation and Bayesian inference, and ρ = 0.54 between analytical error propagation and Bayesian inference. Experience varied by roles. Of respondents who were solely report preparers, 71% had no experience with Bayesian inference, 64% had no experience with Monte Carlo simulation, and 50% had no experience with analytical error propagation. Not surprisingly, respondents who were solely technical assistance providers were more experienced: 29% had no experience with Bayesian inference, 14% had no experience with Monte Carlo simulation, and no one (0%) had no experience with analytical error propagation. Respondents who were both report preparers and technical assistance providers had intermediate experience levels.

**Fig. 4:**
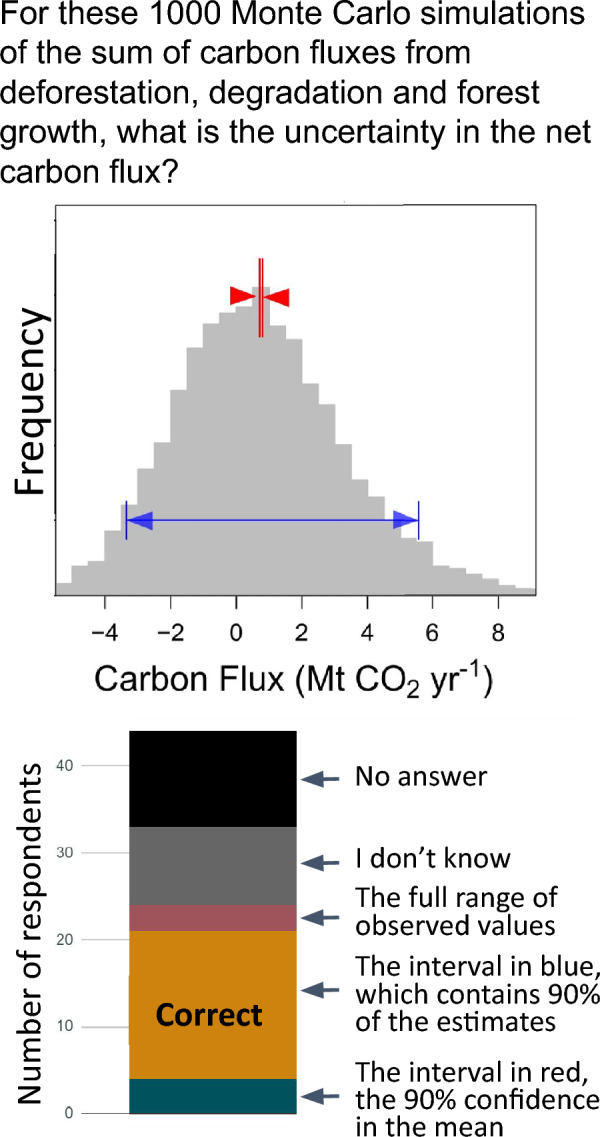
This question tested knowledge of Monte Carlo simulation. Some countries have incorrectly reported the smaller interval (uncertainty in the mean of all outputs, which depends on the number of iterations) rather than the distribution of the outputs

**Fig. 5:**
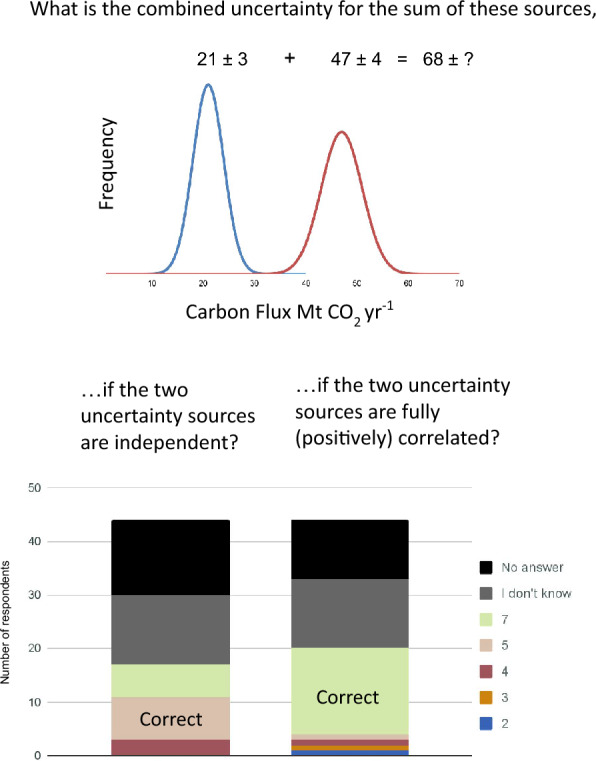
Two questions in the survey tested knowledge of error propagation. Analytical error propagation uses ${\sigma }_{a+b}=\sqrt{{\sigma }_{a}^{2}+{\sigma }_{b}^{2}+2{\sigma }_{a}{\sigma }_{b}}$. Hence, uncertainties of ${\sigma }_{a}=3$ and ${\sigma }_{b}=4$ Mt CO_2_ yr^−1^ combine to ${\sigma }_{a+b}$ = 5 Mt CO_2_ yr^−1^ if they are independent (the two errors being equally likely to be in the same or opposite directions) but ${\sigma }_{a+b}$ = 7 Mt CO_2_ yr^−1^ if they are fully correlated in direction and magnitude

Individuals’ knowledge was explicitly tested by providing three multiple-choice questions that asked about specific concepts related to error propagation. Only 12% of the individuals correctly answered the question about interpretation of Monte Carlo simulation output (Fig. 4). For the questions about the additivity of correlated uncertainty estimates (Fig. 5), only 27% of respondents understood the additivity of independent uncertainty estimates (Fig. 5), with a high percentage of individuals (43%) indicating “don’t know.” Although combining fully correlated uncertainty sources is a more complicated topic, a higher percentage of respondents (48%) correctly answered this question, and a comparable percentage (39%) stated “don’t know.” For the independent errors, the correct answer was 5 $\left(\sqrt{{3}^{2}+{4}^{2}}=\sqrt{25}=5\right)$ and for the correlated errors, the correct answers was 7 $\left(\sqrt{{3}^{2}+{4}^{2}+\left(2\times 3\times 4\right)}=\sqrt{49}=7\right)$. Since the same answer is obtained from 3 + 4 = 7, we cannot be confident that the respondents understood correlation, having tested only the case of full correlation, where this shortcut works. Only 18% of the 33 individuals who answered either of these questions got both of them right.

For all of these knowledge questions, “don’t know” was a common response representing 27% of the responses for Monte Carlo simulation, 39% for sum of correlated uncertainties, and 43% for the sum of independent uncertainties. Responses for the Monte Carlo simulation were correlated with the responses for sum of correlated uncertainty (*p* = 0.05) as were responses for sum of correlated uncertainty and sum of independent uncertainty (*p* = 0.02), but responses for Monte Carlo simulation and sum of independent uncertainty were not correlated (*p* = 0.51).

The responses to the knowledge questions had the expected relationships with experience levels, but the patterns were not significant (*Χ*^*2*^ p ≥ 0.05) which may be due to the small sample size. Only the more experienced respondents correctly answered the Monte Carlo simulation questions (4 respondents, or 19%) versus none of the less experienced respondents. The comparable numbers are 57% and 33% for the additivity of correlated uncertainty estimates and 32% and 18% for the additivity of the independent uncertainty estimates. Not surprisingly, respondents with low experience were more likely to respond “don’t know” and less likely to report an incorrect answer than experienced respondents.

**Fig. 6:**
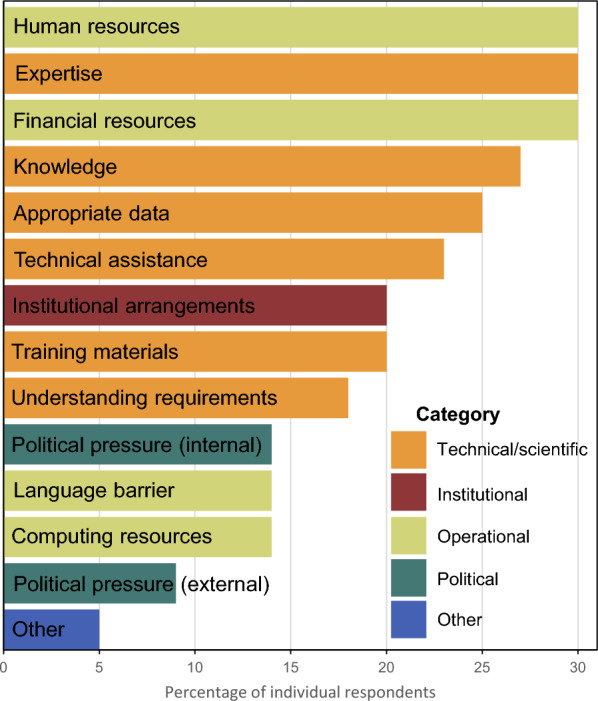
Barriers reported for assessing uncertainty in REDD+ reporting. Percentages include individuals who strongly or somewhat agree that the issue is a barrier using a 5-point Likert scale

**Fig. 7:**
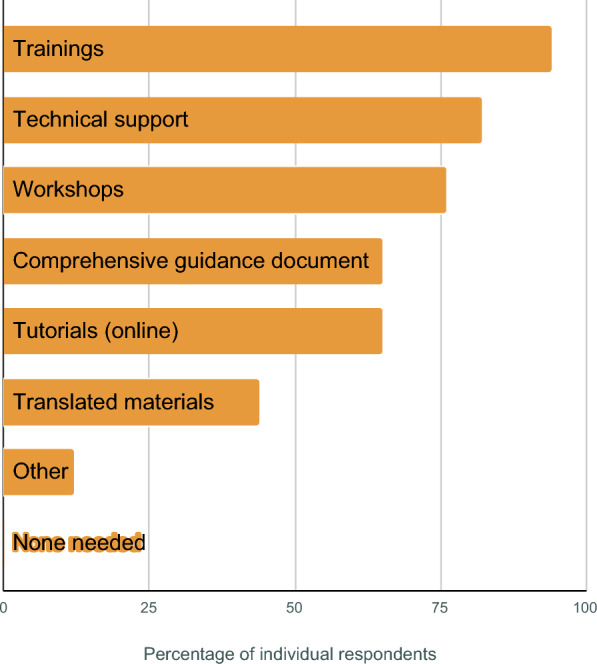
Assistance reported as beneficial for gaining knowledge about improving uncertainty in REDD+ reporting

The desire to improve uncertainty reporting was strong, with all respondents indicating that it was extremely important (52%) or very important (48%), but there are substantial barriers. There was no specific issue that was rated by most individuals as a barrier for improving uncertainty estimates, but the most common were operational issues, including human and financial resources, and technical issues, including expertise, knowledge, appropriate data, and technical assistance (Fig. 6). The most commonly reported desired modes for gaining knowledge to improve uncertainty were trainings, technical support, and workshops (Fig. 7); no individuals indicated that no assistance was needed. The “other” assistance included financial support, a strongly enforced requirement for technical standards, a digital repository of step-by-step methodologies and procedures, and development of improved, species-specific allometric equations.

In response to the open-ended questions, some respondents highlighted the need to establish internationally agreed-upon standardized methodologies that respond to “donor requests on detailed and accurate information” and accompany “the current landscape of incremental requirements by the climate finance mechanisms.” This was also reflected in the respondents rating expertise, knowledge, technical assistance, and training materials as important barriers to uncertainty reporting [21]. These technical barriers were only a part of the barriers reported. Operational barriers (e.g., human and financial resources), surprisingly, appeared to be more important than institutional ones.

Two points were raised in the open-ended responses that address barriers beyond the scope of the reporting teams. Technical assessments of the country reports (e.g., FREL/FRL, REDD+ Technical Annex) are carried out by UNFCCC. One respondent said:

“...[the evaluators] should take special care of uncertainty. Usually their review highlights other things (instruments, transparency, consistency, etc) that are ok but overlook uncertainty. I believe this is in part because of lack of training by evaluators.”

Several respondents addressed the negative perceptions of uncertainty by decision-makers:

“Usually countries’ policy makers in charge of the REDD+ decisions have a black and white approach (absolute accounting) to uncertainty and understand an error as a bad process and a wrong result by the country that should be repeated by scratch [sic].”

## Discussion

Most respondents reported that uncertainty in REDD+ was very or extremely important, but their actual comfort levels and knowledge with specific techniques were generally low. Although uncertainty is not required in reports to the UNFCCC of REDD+ associated emissions, it is recommended for transparency in reporting [22, 23], and reporting is improving over time (Fig. 1). Programs for payment, such as FCPF’s Carbon Fund, generally require uncertainty to be reported, and payments may be lower if uncertainties are high [7, 9]. Because uncertainty factors into the payments made, there is a perverse incentive for countries to omit or underestimate sources of uncertainty [24]. Including more sources of uncertainty makes the confidence intervals wider, giving a false impression that the quality of the estimates is worse [25]. An improved assessment of uncertainties is likely to result in a higher aggregate uncertainty because more sources of error are captured in the uncertainty assessment. Like omitting uncertainty sources, incorrectly calculating uncertainties can reduce their magnitude. For example, some countries have reported uncertainties based on the standard error of Monte Carlo distributions, when they should have used the standard deviation (Fig. 5). Many results-based payment programs (e.g., FPCF) cap the payment deduction for high uncertainties to limit the incentive to underreport.

**Fig. 8:**
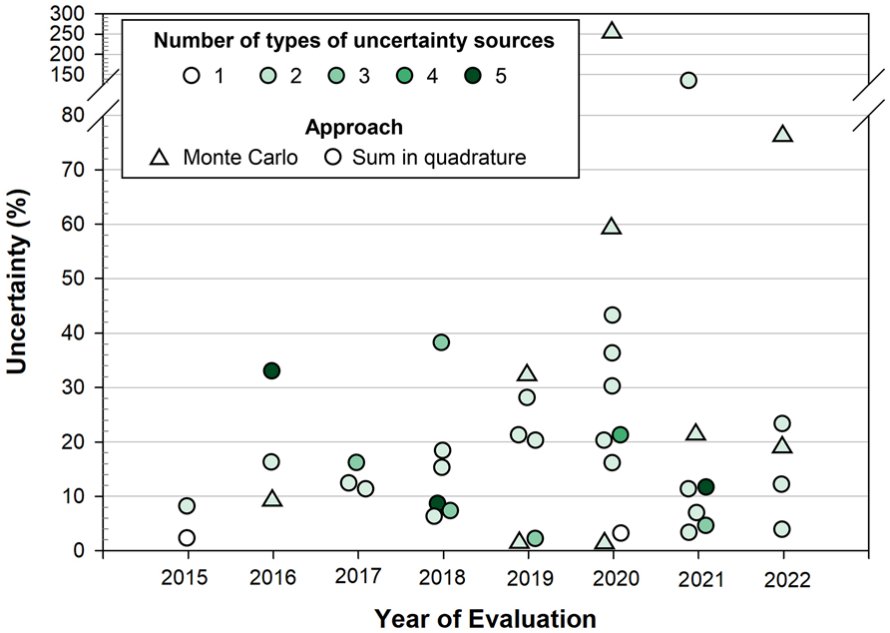
Uncertainties in reference levels of emissions reported by 40 countries that have submitted to the UNFCCC, 5 of them twice. 21 additional countries submitted reports without a combined uncertainty estimate. Colors indicate the number of types of uncertainty sources included, ranging from 1 to 5, namely, tree measurement, allometric models, variability in forest carbon (sampling error), land-use change, and other parameters (carbon fraction and root-to-shoot ratio). Not all countries calculated and propagated uncertainty sources correctly

The quality and completeness of uncertainty reporting was low, both according to our survey respondents and our independent evaluation of country reports submitted to the UNFCCC (Fig. 8). For example, while measurement errors and root-to-shoot ratios were scored as important, very few submissions report them [26]. Sampling error in forest biomass and uncertainties in biomass models are also under-reported relative to their perceived importance. Only errors in activity data were consistently considered important and reported. Activity data have historically been found to contribute the greatest uncertainty to both emissions and emission reductions, and thus financial and technical investments to quantify uncertainty have been focused in this area [27]. Because other sources of uncertainty have rarely been quantified, it is possible that they may be more important. Change in soil carbon storage is highly uncertain and likely important, but since it is not required in REDD+ reporting (except for peatlands under ART-TREES), it was not considered in this survey.

It is not surprising that respondents had greater experience with analytical approaches than with the more advanced Monte Carlo or Bayesian methods to estimate errors in carbon accounting. Early IPCC guidelines [28] emphasized analytical approaches, but Monte Carlo approaches have been required recently by some programs, such as the FCPF or ART-TREES [29, 30]. In a stepwise approach to REDD+ accounting, countries may begin with analytical error propagation, often ignoring correlation among uncertainty sources, and then proceed to Monte Carlo approaches. Bayesian approaches, not yet included as an option by IPCC [24], have yet to be adopted for overall uncertainty in country-level carbon accounting, but they are beginning to be used for detecting land-use change for estimating activity data [31].

Yet, even for analytical error propagation, the experience with error estimation was low: 60% of respondents report little or no experience with these techniques. This is surprising given the fact that many countries have already undergone several submissions of Forest Reference Levels and/or REDD+ emission reductions reports (e.g., as an annex to their biennial update reports) and that 93% of the respondents stated the high importance of reporting uncertainties. This indicates that despite the improvements in capacities within the REDD+ Measuring, Reporting, and Verification (MRV) mechanisms [32], experience in reporting emission reductions is still highly variable [33]. Given that the sustainability of MRV for REDD+ depends heavily on local capacity to understand and apply statistically appropriate estimators and new technologies [34, 35], the results indicate that capacity development in the sector is still greatly needed, even at the level of reporting experts.

Uncertainty reporting is both important and not very well understood, but fortunately there is a desire to increase the reliability of the estimates. REDD+ reporting requires stepwise and consistent improvement in the development of National Forest Monitoring systems, which include reporting [36, 37]. Improvements are indeed ongoing, even if some countries cannot yet access payments for results because of large uncertainties or unconvincing or incomplete methodologies. There is also a need to improve the knowledge and skills of the auditors and experts conducting technical assessment of reported emission reductions. Programs such as Quantifying Uncertainty Estimates and Risk for Carbon Accounting (QUERCA), funded by the USDA Forest Service International Programs, can play an important role in these improvements, in conjunction with technical agencies traditionally providing capacity development in the sector, such as the United Nation’s FAO. Experts from these institutions, using the results from this study, can be instrumental in developing the requested training and workshops and providing technical support. All of these efforts could be tailored to meet the individuals at their current knowledge and experience levels using open and accessible resources. Assessing the efficacy of such efforts may prove useful, and this survey represents a baseline.

## Conclusions

Although few countries thoroughly quantify and document estimates of uncertainty in REDD+ reports [7], our survey results show that most respondents feel it is important. Respondents indicated that current knowledge for quantifying uncertainty is relatively low and there is a desire to improve the estimates. Organizations that fund REDD+ have an interest in improving estimates of uncertainty to increase the transparency and reliability of REDD+ reports. Respondents to this survey indicated a desire for more training and other technical assistance. Future research could further focus on the needs of the people generating the reports and assess the efficacy of potential assistance programs.

Overcoming barriers, whether technical, institutional, or financial, is critical for advancing uncertainty reporting. Countries may benefit from technological improvements in acquiring or analyzing data, such as the use of model-based inference or high-resolution imagery that could reduce uncertainties [38, 39]. Technical support would ideally facilitate integrated methodologies that align the already complex analyses necessary to propagate possible sources of uncertainty while building capacity among people involved in these analyses [37]. These technical and institutional challenges are unattainable without sufficient and sustained financial support from external donors or domestic budgets for forest monitoring, preferably enshrined in national laws [40]. Finally, a fundamental shift in the negative perception and communication of uncertainty is needed by all stakeholders involved, from donor to receiving countries. Knowledge of uncertainty provides opportunities for improvement in decision-making through the maintenance of a wide and flexible range of response options [41], which are fundamental to the development of national forest monitoring systems [40]. We hope that the results of this survey will help guide both countries and donors to prioritize efforts in those areas most needed to address uncertainties in carbon accounting.

### Supplementary Information

Supplementary Material 1.

## Data Availability

The survey instrument is provided in the Supplemental Materials. To maintain the anonymity of the respondents, raw survey results will not be made public.
