# Supplementary material for: Uncertainty in REDD+ carbon accounting: a survey of experts involved in REDD+ reporting
Source: Carbon Balance Manag. 2024 Jul 27;19:22. doi: 10.1186/s13021-024-00267-z (PMC11283695; doi:10.1186/s13021-024-00267-z)
Supplement: Supplementary file 1 — Supplementary Material 1. [file 13021_2024_267_MOESM1_ESM.docx]

QUERCA

# [Welcome]

Q1

Welcome to the Uncertainty in REDD+ Reporting Survey!

Bienvenue dans l’enquête sur l’incertitude dans les rapports REDD+!

¡Bienvenido a la Encuesta sobre la Incertidumbre en los Informes de REDD+!

# [Language]

Q131 Please select your language.

o English (1)

o Français (2)

o Español (3)

# [Country]

Q2 For what country do you work on REDD+ reporting?

If you have worked on REDD+ reporting for multiple countries, please respond for the country that you most recently worked with.

________________________________________________________________

# [Role]

Q3

For this most recent report, what was your role in report preparation?

Check all that apply.

▢ Preparation (1)

▢ Technical assistance (2)

▢ Oversight (3)

▢ Reviewing (4)

Q4 What is the most recent year you were involved in REDD+ reporting?

▼ 2022 (1) ... 2007 (16)

# [General Attitudes Regarding Uncertainty]

Q5

Importance of uncertainty

How important is it for ${Q2/ChoiceTextEntryValue} to report estimates of uncertainty for REDD+ forest carbon accounting?

o Extremely important (5)

o Very important (4)

o Somewhat important (3)

o A little important (2)

o Not at all important (1)

Q6 How comprehensive is the current reporting of carbon accounting uncertainty for ${Q2/ChoiceTextEntryValue}?

Comprehensive means all sources of uncertainty are included.

o All sources of uncertainty are reported (5)

o Most sources of uncertainty are reported (4)

o Some sources of uncertainty are reported (3)

o Few sources of uncertainty are reported (2)

o No sources of uncertainty are reported (1)

o I don't know (9)

Q7

How correct is the current reporting of carbon accounting uncertainty for ${Q2/ChoiceTextEntryValue}?

Correct means quantification and error propagation are correct.

o Uncertainty is highly correct (5)

o Uncertainty is mostly correct (4)

o Uncertainty is moderately correct (3)

o Uncertainty is a little correct (2)

o Uncertainty is not at all correct (1)

o I don't know (9)

Q8 How important, for you personally, is it to improve estimates of uncertainty in REDD+ forest carbon accounting?

o Extremely important (5)

o Very important (4)

o Somewhat important (3)

o A little important (2)

o Not at all important (1)

# [Sources of Error]

Q9 Sources of Error

In the next sections, we will ask about different sources of uncertainty in emissions factors: - Sampling error - Measurement error, for example in tree height and diameter - Error in root-to-shoot ratios - Uncertainty in biomass modelsWe will also ask about uncertainty in activity data.

## [Sampling Error]

Q10 Sampling error in emission factors

Sampling error describes variability across multiple samples, such as forest plots.

Q11 How important, or unimportant, is it to improve quantification of sampling error in REDD+ forest carbon accounting?

o Extremely important (5)

o Very important (4)

o Moderately important (3)

o Slightly important (2)

o Not at all important (1)

Q12 Does ${Q2/ChoiceTextEntryValue} report sampling error in REDD+ forest carbon accounting?

o Yes (1)

o No (0)

o I don't know (9)

Display This Question:

If Does ${q://QID106/ChoiceTextEntryValue} report sampling error in REDD+ forest carbon accounting? = No

Q13 What is the reason(s) for not reporting sampling error in REDD+ forest carbon accounting? Select all that apply.

▢ It's not important (1)

▢ It's not required (2)

▢ Lack of technical skills (3)

▢ Reporting this source increases overall uncertainty (4)

▢ Lack of available data (5)

▢ Lack of financial resources (6)

▢ Other (Please specify): (7) __________________________________________________

Q14 How experienced are you with quantifying sampling error in REDD+ forest carbon accounting?

o Extremely experienced (5)

o Very experienced (4)

o Somewhat experienced (3)

o A little experienced (2)

o Not at all experienced (1)

## [Measurement Error]

Q15 Measurement error

Measurement error includes incorrect measurement by field technicians and uncertainty in measurement tools, such as for tree height or diameter.

Q16 How important, or unimportant, is it to improve quantification of measurement error in REDD+ forest carbon accounting?

o Extremely important (5)

o Very important (4)

o Moderately important (3)

o Slightly important (2)

o Not at all important (1)

Q17 Does ${Q2/ChoiceTextEntryValue} report measurement error in REDD+ forest carbon accounting?

o Yes (1)

o No (0)

o I don't know (2)

Display This Question:

If Does ${q://QID106/ChoiceTextEntryValue} report measurement error in REDD+ forest carbon accounting? = No

Q18 What is the reason(s) for not reporting measurement error in REDD+ forest carbon accounting? Select all that apply.

▢ It's not important (1)

▢ It's not required (2)

▢ Lack of technical skills (3)

▢ Reporting this source increases overall uncertainty (4)

▢ Lack of available data (5)

▢ Lack of financial resources (6)

▢ Other (Please specify): (7) __________________________________________________

Q19 How experienced are you with quantifying measurement error in REDD+ forest carbon accounting?

o Extremely experienced (5)

o Very experienced (4)

o Somewhat experienced (3)

o A little experienced (2)

o Not at all experienced (1)

## [Error in root-to-shoot ratios]

Q20 Error in root-to-shoot ratios

Error in root-to-shoot ratios includes selection of the correct ratios and the error associated with the ratios.

Q21 How important, or unimportant, is it to improve quantification of error in root-to-shoot ratios in REDD+ forest carbon accounting?

o Extremely important (5)

o Very important (4)

o Moderately important (3)

o Slightly important (2)

o Not at all important (1)

Q22 Does ${Q2/ChoiceTextEntryValue} report errors in root-to-shoot ratios in REDD+ forest carbon accounting?

o Yes (1)

o No (0)

o I don't know (9)

Display This Question:

If Does ${q://QID106/ChoiceTextEntryValue} report errors in root-to-shoot ratios in REDD+ forest car... = No

Q23 What is the reason(s) for not reporting errors in root-to-shoot ratios in REDD+ forest carbon accounting? Select all that apply.

▢ It's not important (1)

▢ It's not required (2)

▢ Lack of technical skills (3)

▢ Reporting this source increases overall uncertainty (4)

▢ Lack of available data (5)

▢ Lack of financial resources (6)

▢ Other (Please specify): (7) __________________________________________________

Q24 How experienced are you with quantifying errors in root-to-shoot ratios in REDD+ forest carbon accounting?

o Extremely experienced (5)

o Very experienced (4)

o Somewhat experience (3)

o A little experienced (2)

o Not at all experienced (1)

## [Uncertainty in biomass models]

Q25 Uncertainty in biomass models

Uncertainty in biomass models includes selection of the appropriate allometric model and uncertainty in the model fit.

Q26 How important, or unimportant, is it to improve quantification of uncertainty in biomass models in REDD+ forest carbon accounting?

o Extremely important (5)

o Very important (4)

o Moderately important (3)

o Slightly important (2)

o Not at all important (1)

Display This Question:

If Does ${q://QID106/ChoiceTextEntryValue} report uncertainty in biomass models in REDD+ forest carb... = No

Q28 What is the reason(s) for not reporting uncertainty in biomass models? Select all that apply.

▢ It's not important (1)

▢ It's not required (2)

▢ Lack of technical skills (3)

▢ Reporting this source increases overall uncertainty. (4)

▢ Lack of available data (5)

▢ Lack of financial resources (6)

▢ Other (Please specify): (7) __________________________________________________

Q29 How experienced are you with quantifying uncertainty in biomass models in REDD+ forest carbon accounting?

o Extremely experienced (5)

o Very experienced (4)

o Somewhat experience (3)

o A little experienced (2)

o Not at all experienced (1)

## [Uncertainty in activity data]

Q30 Uncertainty in activity data

Uncertainty in activity data includes uncertainty around land cover change such as deforestation, forest degradation, and reforestation.

Q31 How important, or unimportant, is it to improve quantification of uncertainty in activity data in REDD+ forest carbon accounting?

o Extremely important (5)

o Very important (4)

o Moderately important (3)

o Slightly important (2)

o Not at all important (1)

Q32 Does ${Q2/ChoiceTextEntryValue} report uncertainty in activity data in REDD+ forest carbon accounting?

o Yes (1)

o No (0)

o I don't know (9)

Display This Question:

If Does ${q://QID106/ChoiceTextEntryValue} report uncertainty in activity data in REDD+ forest carbo... = No

Q33 What is the reason(s) for not reporting the uncertainty in activity data?

Choose all that apply.

▢ It's not important (1)

▢ It's not required (2)

▢ Lack of technical skills (3)

▢ Reporting this source increases overall uncertainty. (4)

▢ Lack of available data (5)

▢ Lack of financial resources (6)

▢ Other (Please specify): (7) __________________________________________________

Q34 How experienced are you with quantifying uncertainty in activity data in REDD+ forest carbon accounting?

o Extremely experienced (5)

o Very experienced (4)

o Somewhat experienced (3)

o A little experienced (2)

o Not at all experienced (1)

## [Other Sources of Uncertainty]

Q35

Are there other sources of uncertainty in REDD+ carbon accounting not described above that are important to improve the quantification of?

Please list them below.

________________________________________________________________

# [Experience with Methods]

Q36 The next section is going to ask you about your experience with:

- Monte Carlo simulation (IPCC Approach 2)

- Analytical error propagation (IPCC Approach 1)

- Bayesian inference

Q37

Monte Carlo simulation

Monte Carlo simulation is a numerical technique that generates many estimates of a calculation with random samples of the variables. Bootstrapping is an application of the Monte Carlo technique.

Q38

How experienced are you with Monte Carlo simulation techniques for estimating uncertainty in REDD+ forest carbon accounting analyses?

o Extremely experienced (5)

o Very experienced (4)

o Somewhat experienced (3)

o A little experienced (2)

o Not at all experienced (1)

Q39

Analytical error propagation

Analytical error propagation uses mathematical techniques for combining uncertainty, including Gaussian error propagation, method of moments, or IPCC Approach I.

Q40

How experienced are you with using analytical error propagation for estimating uncertainty in REDD+ forest carbon accounting analyses?

o Extremely experienced (5)

o Very experienced (4)

o Somewhat experienced (3)

o A little experienced (2)

o Not at all experienced (1)

Q41

Bayesian techniques

Bayesian inference is a method of statistical inference in which Bayes' theorem is used to update the probability for a hypothesis as more evidence or information becomes available.

Q42

How experienced are you with using Bayesian techniques for estimating uncertainty in REDD+ forest carbon accounting analyses?

o Extremely experienced (5)

o Very experience (4)

o Somewhat experienced (3)

o A little experienced (2)

o Not at all experience (1)

# [Barriers & Opportunities]

Q43

Barriers to improving uncertainty analyses

The following questions are about barriers to improving uncertainty analyses in forest carbon accounting and reporting.

Q44

To what extent do you agree or disagree with the following barriers to improving uncertainty analyses in ${Q2/ChoiceTextEntryValue}?

Strongly

agree (5) Somewhat

agree (4) Neither agree

nor disagree (3) Somewhat

disagree (2) Strongly

disagree (1)

Lack of knowledge of the appropriate techniques (Q47_1) o o o o o

Lack of training materials (Q47_2) o o o o o

Lack of expertise to implement the techniques (Q47_3) o o o o o

Lack of technical assistance (Q47_4) o o o o o

Lack of human resources (Q47_5) o o o o o

Lack of financial resources (Q47_6) o o o o o

Lack of appropriate data (Q47_7) o o o o o

Lack of computing resources (Q47_8) o o o o o

Lack of understanding of REDD+ requirements (Q47_9) o o o o o

Language barrier (Q47_10) o o o o o

Institutional arrangements (Q47_11) o o o o o

Political pressure from my country (Q47_12) o o o o o

Political pressure from other countries (Q47_13) o o o o o

Improvement is not necessary - uncertainty analyses are adequate (Q47_14) o o o o o

Other (Please specify): (Q47_15) o o o o o

Q45

What assistance, if any, would help to improve estimates of uncertainty in REDD+ forest carbon accounting that you report?

Select all that apply.

▢ Trainings (1)

▢ Workshops (2)

▢ Tutorials on the internet (3)

▢ A comprehensive guidance document (4)

▢ Translation of materials to another language (please specify which language): (5) __________________________________________________

▢ Technical support (6)

▢ Other (please specify): (7) __________________________________________________

▢ ⊗No assistance needed (8)

# [Knowledge]

Q48

Knowledge of uncertainty

The following three questions test knowledge of uncertainty calculation. We will use your answers to help address specific gaps in knowledge.

Q49


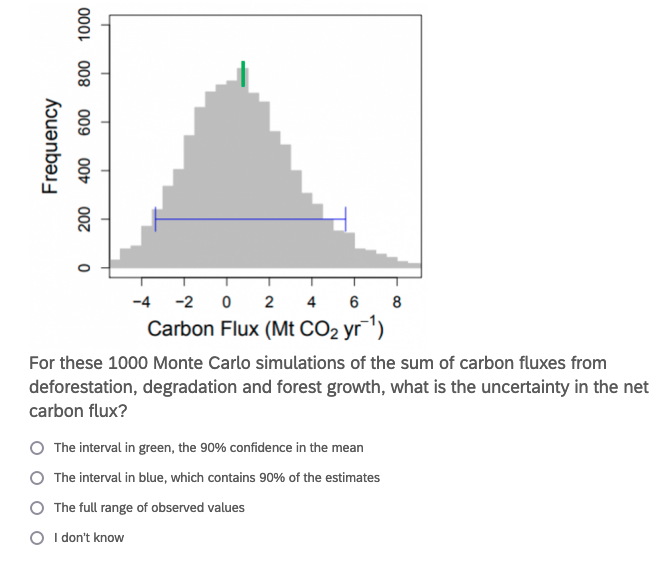


For these 1000 Monte Carlo simulations of the sum of carbon fluxes from deforestation, degradation and forest growth, what is the uncertainty in the net carbon flux?

o The interval in green, the 90% confidence in the mean (1)

o The interval in blue, which contains 90% of the estimates (2)

o The full range of observed values (3)

o I don't know (9)

Q50 In the following figures, two uncertainty sources are perfectly (positively) correlated, with means and 90% confidence intervals of 21 Mt CO2 ± 3 Mt CO2 and 47 Mt CO2 ± 4 Mt CO2.


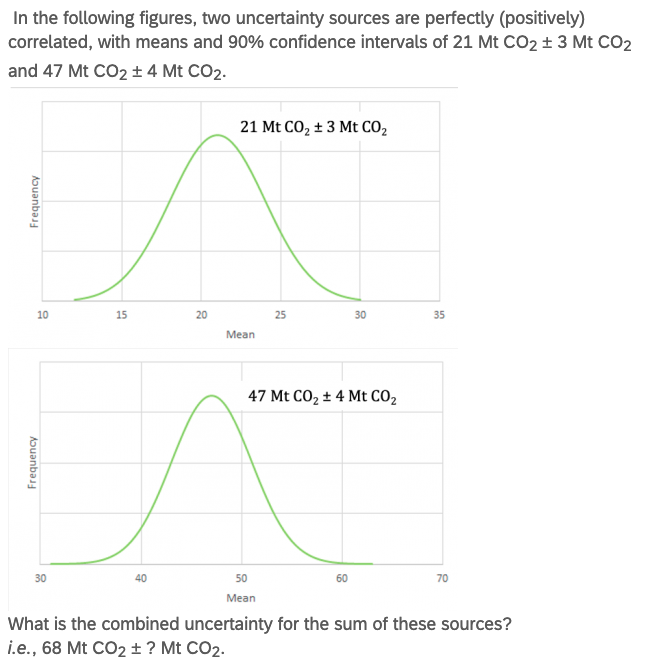


What is the combined uncertainty for the sum of these sources? i.e., 68 Mt CO2 ± ? Mt CO2.

o 2 (1)

o 3 (2)

o 4 (3)

o 5 (4)

o 7 (5)

o I don’t know (9)

Q51 In the following figure, two uncertainty sources are independent with mean and 90% confidence intervals of 21 Mt CO2 ± 3 Mt CO2 and 47 Mt CO2 ± 4 Mt CO2. These are identical to the previous question, except the uncertainty sources are independent.


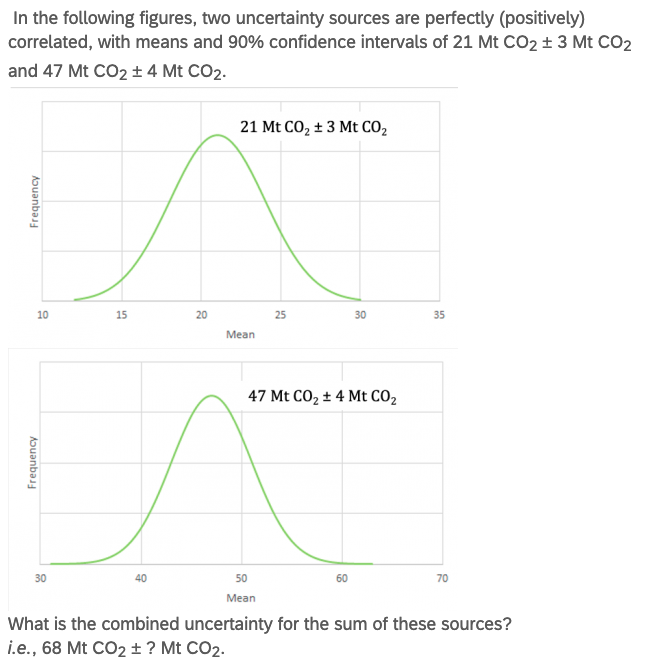


What is the combined uncertainty for the sum of these sources?

i.e., 68 Mt CO2 ± ? Mt CO2.

o 2 (1)

o 3 (2)

o 4 (3)

o 5 (4)

o 7 (5)

o I don’t know (9)

# [Background - Country]

Q52 Country background

For the most recent report for ${Q2/ChoiceTextEntryValue}, who prepared the REDD+ uncertainty estimates?

Check all that apply.

▢ Government employees (1)

▢ Consultants (2)

▢ Donor agency (3)

▢ Research institutions (4)

▢ Other (Please specify): (5) __________________________________________________

▢ ⊗I don't know (6)

Q53 For the most recent report for ${Q2/ChoiceTextEntryValue}, who decided whether to use Monte Carlo simulation (Approach 2) or analytical methods (Approach 1) for error propagation?

Check all that apply.

▢ Government employees (1)

▢ Consultants (2)

▢ REDD+ officers (3)

▢ Required by donor agency (4)

▢ Other (Please specify): (5) __________________________________________________

▢ ⊗I don't know (6)

Q54 For the most recent report for ${Q2/ChoiceTextEntryValue}, who decided what sources of uncertainty in REDD+ forest carbon accounting were reported?

Check all that apply.

▢ Government employees (1)

▢ Consultants (2)

▢ REDD+ officers (3)

▢ Required by donor agency (4)

▢ Other (Please specify): (5) __________________________________________________

▢ ⊗I don't know (6)

Q55 How, if at all, is the report reviewed before it becomes the official report for ${Q2/ChoiceTextEntryValue}?

Check all that apply.

▢ Internal REDD+ officers review it (1)

▢ Consultants review it (2)

▢ A national technical stakeholders group reviews it (3)

▢ External experts review it (4)

▢ Public review and comment (5)

▢ Other (Please specify): (6) __________________________________________________

▢ ⊗It's not reviewed (7)

▢ ⊗I don't know (8)

# [Background - Respondent]

Q56 Background information about you

Q57 Other than ${Q2/ChoiceTextEntryValue}, which country's or countries' carbon accounting for REDD+ reports have you contributed to?

Select all that apply.

▢ ⊗No other countries (1)

▢ Argentina (2)

▢ Bangladesh (3)

▢ Belize (4)

▢ Bhutan (5)

▢ Brazil (6)

▢ Burkina Faso (7)

▢ Cambodia (8)

▢ Central African Republic (9)

▢ Chile (10)

▢ Colombia (11)

▢ Côte D'Ivoire (12)

▢ Costa Rica (13)

▢ Democratic Republic of Congo (14)

▢ Dominican Republic (15)

▢ Ecuador (16)

▢ EI Salvador (17)

▢ Equatorial Guinea (18)

▢ Ethiopia (19)

▢ Fiji (20)

▢ Ghana (21)

▢ Guatemala (22)

▢ Guinea-Bissau (23)

▢ Guyana (24)

▢ Honduras (25)

▢ India (26)

▢ Indonesia (27)

▢ Kenya (28)

▢ Lao PDR (29)

▢ Liberia (30)

▢ Madagascar (31)

▢ Malawi (32)

▢ Malaysia (33)

▢ Mexico (34)

▢ Mongolia (35)

▢ Mozambique (36)

▢ Myanmar (37)

▢ Nepal (38)

▢ Nicaragua (39)

▢ Nigeria (40)

▢ Pakistan (41)

▢ Panama (42)

▢ Papua New Guinea (43)

▢ Paraguay (44)

▢ Peru (45)

▢ Solomon Islands (46)

▢ Sri Lanka (47)

▢ Sudan (48)

▢ Suriname (49)

▢ Tanzania (50)

▢ Togo (51)

▢ Uganda (52)

▢ Vietnam (53)

▢ Zambia (54)

▢ Vanuatu (55)

Q58 When you most recently worked on the REDD+ report for ${Q2/ChoiceTextEntryValue}, what best describes the organization that you worked for?

o Government (1)

o International organization (2)

o Non-governmental organization (NGO) (3)

o Independent consultant (4)

o Private company (5)

o Academic or research institution (6)

o Other (please specify): (7) __________________________________________________

Q59

What other types of organizations, if any, have you worked for while doing carbon accounting for REDD+ reporting?

Please check all that apply.

▢ Government (1)

▢ International organization (2)

▢ Non-governmental organization (NGO) (3)

▢ Independent consultant (4)

▢ Private company (5)

▢ Academic or research institution (6)

▢ Other (please specify): (7) __________________________________________________

▢ ⊗No other types of organizations (8)

Q60 How many years of experience do you have with REDD+ reporting?

________________________________________________________________

Q61 What is your highest level of education?

o High school (1)

o Some college (2)

o Bachelors degree (3)

o Some graduate school (4)

o Masters degree (5)

o Doctorate (6)

Q62 In what topic areas did you receive your degree(s)?

________________________________________________________________

Q63 In your opinion, how can countries be encouraged to correctly report carbon accounting uncertainty?

________________________________________________________________

________________________________________________________________

________________________________________________________________

________________________________________________________________

________________________________________________________________

Q64 In your own words, why is uncertainty reporting for carbon accounting important or not important?

________________________________________________________________

________________________________________________________________

________________________________________________________________

________________________________________________________________

________________________________________________________________

Q65

Final question

Do you have any comments you want to share? If so, please add them below.

________________________________________________________________

________________________________________________________________

________________________________________________________________

________________________________________________________________

________________________________________________________________
